# The Interaction of Temozolomide with Blood Components Suggests the Potential Use of Human Serum Albumin as a Biomimetic Carrier for the Drug

**DOI:** 10.3390/biom10071015

**Published:** 2020-07-09

**Authors:** Marta Rubio-Camacho, José A. Encinar, María José Martínez-Tomé, Rocío Esquembre, C. Reyes Mateo

**Affiliations:** Instituto e investigación, Desarrollo e Innovación en Biotecnología Sanitaria de Elche (IDiBE), Universidad Miguel Hernández (UMH), E-03202 Elche, Spain; marta.rubioc@umh.es (M.R.-C.); jant.encinar@umh.es (J.A.E.); mj.martinez@umh.es (M.J.M.-T.)

**Keywords:** temozolomide (TMZ), bloodstream components interaction, human serum albumin (HSA), alpha-1-acid glycoprotein (AGP), model biomembranes, molecular docking

## Abstract

The interaction of temozolomide (TMZ) (the main chemotherapeutic agent for brain tumors) with blood components has not been studied at the molecular level to date, even though such information is essential in the design of dosage forms for optimal therapy. This work explores the binding of TMZ to human serum albumin (HSA) and alpha-1-acid glycoprotein (AGP), as well as to blood cell-mimicking membrane systems. Absorption and fluorescence experiments with model membranes indicate that TMZ does not penetrate into the lipid bilayer, but binds to the membrane surface with very low affinity. Fluorescence experiments performed with the plasma proteins suggest that in human plasma, most of the bound TMZ is attached to HSA rather than to AGP. This interaction is moderate and likely mediated by hydrogen-bonding and hydrophobic forces, which increase the hydrolytic stability of the drug. These experiments are supported by docking and molecular dynamics simulations, which reveal that TMZ is mainly inserted in the subdomain IIA of HSA, establishing π-stacking interactions with the tryptophan residue. Considering the overexpression of albumin receptors in tumor cells, our results propose that part of the administered TMZ may reach its target bound to plasma albumin and suggest that HSA-based nanocarriers are suitable candidates for designing biomimetic delivery systems that selectively transport TMZ to tumor cells.

## 1. Introduction

**Scheme 1:**
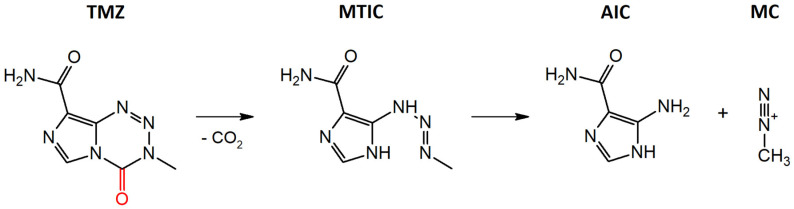
TMZ hydrolysis to 5-(3-methyltriazen-1-yl) imidazole-4-carboxamide (MITC), 5-amino-imidazole-4-carboxamide (AIC) and methyldiazonium ion (MC) in aqueous solution.

Glioblastoma multiforme (GBM) is the most frequent and aggressive primary malignant brain tumor in adults. Current treatment options are multimodal and include surgical resection, radiation and chemotherapy [1]. The prodrug temozolomide (TMZ), a bis(imidazotetrazine) heterocycle, is the main chemotherapeutic agent used [2,3,4]. Combined with radiation therapy, it contributes to extend the survival period and improves the patient’s quality of life [5]. TMZ is rapidly and completely absorbed from the gastrointestinal tract after oral administration. After this, the compound is spontaneously hydrolyzed at a physiological pH to the highly unstable metabolite 5-(3-methyltriazen-1-yl) imidazole-4-carboxamide (MTIC), which rapidly degrades to 5-amino-imidazole-4-carboxamide (AIC) and methyldiazonium ion (MC) (Scheme 1) [6]. This highly reactive cation interacts with the DNA, causing its methylation, and this consequently results in cell death [7].

TMZ requires continuous administration due to its low solubility and specificity, as well as short half-life in blood plasma (≈1.8 h) [3,6]. As a result of prolonged therapy, the drug causes important side effects which results in many cases in the need to interrupt the treatment. In addition, the continuous administration of TMZ gives rise to various resistance mechanisms, being O6-methylguanine-DNA methyltransferase (MGMT), the most important contributor to TMZ chemoresistance [5,8,9,10,11,12,13]. The efficacy of TMZ is also reduced, due to its limited access through the biological barriers (blood-brain barrier and tumor cell membranes) that the drug has to cross to reach the target site [14,15,16,17]. In this regard, Ramalho et al. recently reported that the affinity of TMZ and MTIC for biomembrane models that simulate those of GBM and brain capillary endothelial cells is low [18]. Besides, the efficiency of TMZ may also be limited by the existence of interactions with blood and plasma components whose magnitude significantly affects the concentration of available drug in vivo. Interactions that are too strong would not be beneficial for the distribution of free TMZ, because the drug could be irreversibly retained [19,20]. In contrast, a moderate association to certain components, especially those with a long blood half-life, have the advantage of stabilizing and favoring drug transport, facilitating their arrival to the target site [21]. In this sense, Baker et al. characterized the absorption, metabolism, and excretion of ^14^C-TMZ orally administrated to adult patients [22]. In the study, they determined the extent of TMZ binding to total plasma proteins through an ultrafiltration procedure, finding that ≈15% of the TMZ administered is bound to the protein fraction. However, in spite of the biological importance of this molecule, to our knowledge, no work has been reported which explores, at the molecular level, the interaction of TMZ with the most abundant blood components. These studies are necessary, not only to further understand the bioavailability of the drug, but also to design biomimetic drug-delivery systems based on endogenous components; a strategy that has attracted much attention in recent years [23,24,25].

Blood is a combination of plasma and cells (erythrocytes comprising approx. 95% of the total cellular fraction in the blood). Erythrocytes have a long half-life in circulation (80–130 days) and their membrane can contribute to solubilize drugs, preventing their delivery when the association is strong or facilitating their transport to the target site if they are reversibly retained. In fact, complexation of erythrocytes with drugs is one of the most promising therapeutic alternative approaches for the administration of drugs, especially those that are toxic or rapidly eliminated through kidneys and liver [26,27].

Plasma is an aqueous solution containing proteins, organic molecules and minerals. Human serum albumin (HSA) is the most abundant protein in plasma, which accounts for approximately 65 % of the total proteins, corresponding to a concentration of ≈600 μM. It is a highly soluble and stable globular protein consisting of 585 amino acids, composed of a single polypeptide with three α-helical domains (I-III), each containing two subdomains (A and B) which form several hydrophobic pockets throughout the macromolecule [28]. This multidomain structure allows the protein to reversibly bind and transport a wide variety of exogenous and endogenous compounds in the bloodstream, such as fatty acids, metabolites, nutrients and drugs [29,30,31]. Another interesting property of HSA is its exceptional long half-life in serum (≈18–19 days). Therefore, from a drug delivery perspective, this protein is an ideal carrier because of its combination of longevity and stability [32,33,34,35,36]. In addition, it has been reported that albumin-binding receptors (e.g., SPARC and gp60) are overexpressed on tumor cells and tumor vessel endothelium to increase the uptake of nutrients necessary for growth [37,38]. In consequence, HSA can act as a molecular carrier to selectively transport and accumulate anticancer drugs in target tumor tissues and cells [32,33,34,35,39]. 

Another important protein in blood is alpha-1-acid glycoprotein (AGP), composed of a single polypeptide chain of 181 amino acids. The concentration of AGP in plasma can vary considerably. In healthy subjects, it ranges from 10 to 20 μM, but several inflammatory states and pathological conditions can elevate its concentration up to 3- or 4-fold [40]. It is an all-β protein with high β -sheet content, and its overall fold consists of eight antiparallel strands, forming a barrel-type structure with one central hydrophobic pocket [28]. Similarly to HSA, the binding and transport of a wide variety of compounds, including drugs, is one of the functions of AGP. Therefore, the estimation of drug-AGP affinities is also essential for the design of drug dosage forms [41]. 

The approach taken in this work aims to gain insight on the binding of TMZ with different blood components at a molecular level, and to use this information to better understand how the drug travels through the bloodstream, as well as to propose biomimetic alternatives to improve the efficiency of the drug. With this aim, we first investigated the affinity of TMZ for the lipid components of blood cells by using artificial lipid bilayers that model the external membrane of erythrocytes. The use of these model membranes to simulate the complexity of natural cell membranes is very useful when studying the interaction of drugs in a controlled manner [42,43]. Furthermore, we explored the interaction of TMZ with the plasma proteins HSA and AGP. Changes in the intrinsic fluorescence of these proteins induced by TMZ were mainly used to estimate the binding affinity and other thermodynamic parameters of the interaction. The stability of the drug was also monitored after interaction with the blood components. In addition, molecular docking and dynamics simulations were applied to check the results obtained from experimental studies, and to evaluate the dynamics and stability of the TMZ-HSA complex. Possible binding sites, interacting amino acids and the position/orientation of TMZ in the protein were identified from these methods.

## 2. Materials and Methods

### 2.1. Reagents

Temozolomide (TMZ) was obtained from Tokyo Chemical Industry, TCI (Tokyo, JP). Human serum albumin (HSA) and alpha-1-acid glycoprotein (AGP) were purchased from Sigma Aldrich (St. Louis, MO). The synthetic phospholipids 1,2-dimyristoyl-sn-glycero-3-phosphocholine (DMPC), egg 1,2-diacyl-sn-glycero-3-phosphocholine (EPC) and cholesterol (Chol) were from Sigma-Aldrich (St. Louis, MO), and were used as received. The fluorescent probes 2-dimethylamino-6-lauroylnaphtalene (Laurdan) and 1-6-diphenylhexatriene (DPH) were obtained from Molecular Probes (Eugene, OR), and dissolved in ethanol (3.77 × 10^−4^ M) and dimethylformamide (DMF) (1 × 10^−3^ M), respectively. Water was twice distilled in all-glass apparatus and deionized using Milli-Q equipment (Millipore, Madrid, ES). A phosphate buffer (50 mM, 0.1 M NaCl, pH 7.4) and an acetate buffer (100 mM, pH 5.0) were prepared with deionized doubly distilled water. All other compounds were of analytical or spectroscopic reagent grade. 

### 2.2. LUVs Formation 

Chloroform solutions containing the synthetic phospholipid DMPC, EPC or EPC:Chol (3:1) were first dried by evaporation under a dry nitrogen gas stream. Multilamellar vesicles (MLVs) were formed by resuspending the dried mixture of lipids in a certain volume of phosphate buffer (50 mM, 0.1 M NaCl, pH 7.4), acetate buffer (100 mM, pH 5.0), or deionized doubly distilled water, to reach the required final concentration of 0.5 mM. The vesicle suspension was then heated at a temperature above the phase transition of the phospholipid and vortexed several times. Large unilamellar vesicles (LUVs) with a mean diameter of ≈100 nm were prepared from these MLVs by pressure extrusion through 0.1 µm polycarbonate filters (Nucleopore, Cambridge, MA, USA). All samples were used on the same day that they were prepared.

### 2.3. Labeling of LUVs and Fluorescence Measurements

A few microliters from stock solutions of the probes Laurdan or DPH were added to the LUVs suspension, and allowed to stabilize around 30 min at 30 °C before measurement. The lipid-to-probe ratio was 1:400 for DPH and 1:250 for Laurdan, in molar terms.

### 2.4. Absorption and Fluorescence Experiments

The UV absorption measurements were carried out using a UV-2700 Shimadzu spectrophotometer (Tokyo, JP) at 25 °C and/or 37 °C, by using an AccuBlock^TM^ Digital Dry Bath (Labnet International, Inc. Global, Edison, NJ, USA). Samples were placed in 0.2 cm path length quartz cuvettes. For the lipid-binding experiments, spectra were corrected by subtracting the light scattered by LUVs of identical composition and concentration, but in the absence of TMZ. Fluorescence measurements were carried out in a PTI-QuantaMaster Spectrofluorometer (Birmingham, NJ, USA), interfaced with a Peltier, except fluorescence anisotropy experiments, which were performed in a Cary Eclipse Spectrofluorometer (Varian) fitted with thin polarizers. Samples were placed in 1 cm path length quartz cuvettes. Background intensities were always checked and subtracted from the sample when it was necessary. The fluorescence emission spectra of Laurdan within LUVs in the absence and presence of TMZ were obtained between 405 and 550 nm, below and above the gel-fluid transition temperature of the lipid. Samples were excited at 390 nm to minimize the absorption of TMZ. 

The fluorescence emission spectra of HSA and AGP were recorded between 290 and 400 nm upon excitation at 280 nm, to minimize TMZ absorption. The inner filter effect correction in the fluorescence data was made using the following equation, as described by Lakowicz [44]:

I *cor* = I *obs* 10 ^(A*ex* + A*em*)/2^ (1)

where I*cor* and I*obs* are the corrected and the measured fluorescence intensities, respectively. A*ex* and A*em* represent the differences in the absorbance values of the protein solutions observed by the addition of TMZ at the excitation (280 nm) and the emission (290–400 nm) wavelengths, respectively. 

### 2.5. Fluorescence Anisotropy Measurements

The steady-state anisotropy <r> of DPH, incorporated in LUVs, in the absence and presence of TMZ was obtained as a function of temperature from Equation (2), measuring the vertical and horizontal components of the fluorescence emission with excitation polarized vertically.

< r > = (I _VV_ − GI_VH_)/(I _VV_ + 2GI_VH_) (2)

The G factor (G = I_HV_/I_HH_) corrects for the transmissivity bias introduced by the detection system. Samples were excited at 373 nm (slit width, 5 nm), and the polarized emission was detected at 440 nm. 

### 2.6. Quenching Experiments

The fluorescence emission of HSA (at 15 and 25 °C) and AGP (at 25 °C) was obtained in the absence and presence of different concentrations of TMZ, and the decrease in fluorescence intensity was analyzed according to the Stern–Volmer equation [45]:

I_0_/I = 1 + *K*_SV_[Q] = 1+ *k*_q_τ_0_ [Q] (3)

where, I_0_ and I correspond to the calculated area under the corrected fluorescence spectrum in the absence and presence of quencher, respectively, *K*_SV_ is the Stern–Volmer quenching constant, [Q] is the quencher concentration (TMZ), *k*_q_ is the bimolecular rate constant of the quenching reaction and τ_0_ is the average integral fluorescence life time of fluorophore (tryptophan) which is assumed to be 6.4 ns [46].

The affinity constant *K*a was obtained from the quenching data, using Equation (4) [47]

∆I = I_0_ − I = (I_0_ − I_C_) ([TMZ]/(1/*K*a +[TMZ])) (4)

where, Ic is the fluorescence of the fully complexed protein.

Thermodynamic parameters ∆H (enthalpy change), ∆S (entropy change) and ∆G (free energy change) were determined by the Van’t Hoff equation:

Ln(*K*a) = −∆G/RT = (−∆H/RT) + (∆S/R) (5)

where R is the gas constant (8.3145 J mol^−1^ K^−1^) and T is the absolute temperature (K).

### 2.7. Energy Transfer Experiments

For energy transfer experiments, the TMZ was used as an acceptor of the HSA and AGP tryptophan excitation. The data analysis was carried out using the Förster equations [48]. As for quenching experiments, samples were excited at 280 nm to minimize TMZ absorption, and the emission intensity was measured as the area of the emission spectrum, calculated from 290–400 nm. The Förster radius for energy transfer R_0_ was determined from Equation (6):

R_0_ = 0.2108 [k^2^ Φ_F_ n^−4^*J*] ^(1/6)^ (6)

where *J* is the overlap integral, which was evaluated by the integration described in Equation (7), between the normalized donor emission spectrum, I(λ), and the acceptor absorption spectrum, ε(λ) (in M^−1^ cm^−1^ units), as defined by:

J = ∫_0_^∞^ I(λ) ε(λ) λ^4^ dλ (7)

In Equations (6) and (7), k^2^ is the orientation factor (2/3 for random orientation), Φ_F_ is the quantum yield of the donor in the absence of acceptor (0.1 and 0.07 for HSA and AGP respectively) and n is the refractive index of the medium (1.425). R_0_ is expressed in Å and λ, the wavelength, in nm.

### 2.8. Circular Dichroism Experiments

CD measurements of HSA and TMZ were carried out with a Jasco spectropolarimeter, model J-810 (JASCO, Easton, MD), interfaced with a PTC-423S/15 Peltier-type cell holder for temperature control. Spectra were collected at 25 °C with a scan speed of 50 nm per min, response time of 4 s, and a bandwidth of 1 nm. For each spectrum, four scans were accumulated and averaged to improve the signal-to-noise ratio. Spectra were recorded from 250 to 200 nm, using 0.1 cm quartz cells. HSA concentration was kept constant (3 µM), while each TMZ concentration was varied (0, 20 and 200 µM). A baseline was taken under the same conditions as those used for the sample and subtracted from each spectrum. 

### 2.9. Molecular Docking Simulations

Human serum albumin (HSA) (UniProt code: P02768, PDB code: 1AO6) crystallographic structures were obtained from the Research Collaboratory for Structural Bioinformatics (RCSB). The Protein Data Bank (PDB) was used for the molecular docking of TMZ and dynamic simulation (100 ns) purposes. The crystallographic structures of HSA linked to different antineoplastic drugs [33] have also been used: 9 aminocamptothecin (4L8U), camptothecin (4L9K), bicalutamide (4LA0), idarubicin (4LB2), teniposide (4L9Q) and etoposide (4LB9). The specific edition of protein structures was made using PyMol software (PyMOL Molecular Graphics System, v2.3.3 Schrödinger, New York, NY, USA, LLC, at http://www.pymol.org/) without further optimization. Molecular docking experiments were carried out using YASARA structure v19.9.17 software, executing the AutoDock 4 algorithm with AMBER99 as a force field [49,50,51]. Before carrying out the molecular docking experiments, any molecule present in the initial structures was removed (inhibitors, ions, water, etc.) The YASARA pH command was set to 7.4, also when running the molecular dynamics simulations (see below). The structure of TMZ (PubChem CID: 5394) was obtained from the National Center for Biotechnology Information (NCBI) PubChem database (http://www.ncbi.nlm.nih.gov/pccompound). A total of 999 flexible docking runs were set and clustered (7 Å) around the ligand binding domain cavity, i.e., two complexed compounds belonged to different clusters if the ligand root-mean-square deviation of their atomic positions was greater than a minimum of 7 Å around certain hot spot conformations [52]. The YASARA software calculated the Gibbs free energy variation (∆G, J mol^−1^), with more positive energy values indicating stronger binding. To facilitate their understanding, the YASARA software-calculated ∆G values as positive were arranged with a negative value in Supporting Information Table S1. To calculate this parameter, Autodock Vina uses a force field scoring function that considers the strength of electrostatic interactions, hydrogen bonding between all the atoms of the two binding partners, in the complex, intermolecular van der Waals forces, and solvation and entropy contributions [53]. The ligand-protein interactions have been detected with the protein–ligand interaction profiler (PLIP) algorithm [54].

### 2.10. Molecular Dynamics Simulations

YASARA structure v19.9.17 was used for all the MD simulations, with AMBER14 as a force field. The simulation cell was allowed to include 20 Å surrounding the protein, and filled with water at a density of 0.997 g mL^−1^. Initial energy minimization was carried out under relaxed constraints using steepest descent minimization. Simulations were performed in water at constant pressure-constant temperature (25 °C) conditions. To mimic physiological conditions, counter ions were added to neutralize the system; Na^+^ or Cl^−^ were added in the replacement of water to give a total NaCl concentration of 0.9 %, and pH was maintained at 7.4. Hydrogen atoms were added to the protein structure at the appropriate ionizable groups according to the calculated p*K*a in relation to the simulation pH (i.e., a hydrogen atom will be added if the computed pKa is higher than the pH). The p*K*a was computed for each residue according to the Ewald method [55]. All simulation steps were run by a preinstalled macro (md_run.mcr) within the YASARA suite. Data were collected every 100 ps. The molecular mechanics/Poisson–Boltzmann surface area (MM/PBSA) was implemented with the YASARA macro md_analyzebindenergy.mcr, to calculate the binding free energy with the solvation of the ligand, complex, and free protein, as previously described [52,56,57].

## 3. Results and Discussion

### 3.1. Stability of TMZ in Aqueous Media

**Figure 1:**
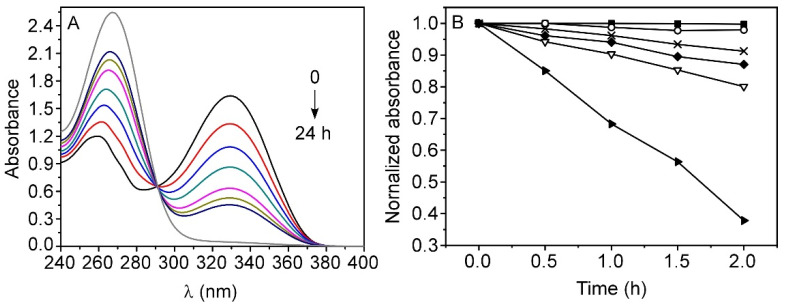
(**A**) Absorption spectra of temozolomide (TMZ) in phosphate buffer pH 7.4 at 37 °C as a function of time up to 24 h. (**B**) Time-dependent degradation of TMZ represented as normalized absorption at major peak (329 nm), under different conditions: acetate buffer (pH 5.0) at 25 (black square) and 37 °C (cross), water at 25 (circle) and 37 °C (triangle) and sodium phosphate buffer pH 7.4 at 25 °C (black diamond) and 37 °C (black triangle).

As described in the Introduction section, TMZ undergoes hydrolytic ring opening under neutral or alkaline conditions to yield the open chain triazene MTIC, which rapidly degrades to AIC. The hydrolytic stability of TMZ can be monitored by UV−VIS spectroscopy, thanks to the different absorption bands of TMZ and AIC. Figure 1A shows the absorption spectrum from 240 to 400 nm, of a freshly prepared solution of TMZ (150 µM) in phosphate buffer, pH 7.4. The spectrum displays two well-resolved bands with a main peak at 329 nm and a less intense peak at 258 nm. The sample was incubated at 37 °C and spectra were recorded over time, at predetermined intervals, up to 24 h. Results show a clear decrease in the intensity of the major absorbance peak (329 nm). In addition, the second absorbance peak (258 nm) becomes obscured by the appearance of a new absorption band at 265 nm, attributed to the amide group of AIC, that evidences the rapid decomposition of TMZ under physiological conditions [6] (Figure 1A). Similar experiments were performed for TMZ in phosphate buffer at 25 °C and in acetate buffer, pH 5.0, or deionized doubly distilled water, at 25 °C and 37 °C (Figure 1B). Results show that TMZ dissolved in water and acetate buffer remains practically unaltered at 25 °C, after 2 h of preparation. For TMZ in phosphate buffer at 25 °C, although at a lesser extent than for the same buffer at 37 °C, degradation is still considerable, denoting the intrinsic instability of the free drug at physiological pH. These observations confirm that temperature accelerates the degradation process of TMZ, and that the drug is much more stable against hydrolysis in acidic media than when dissolved in phosphate buffer, in agreement with previous reports [6]. 

### 3.2. Interaction of TMZ with Model Membranes

**Figure 2:**
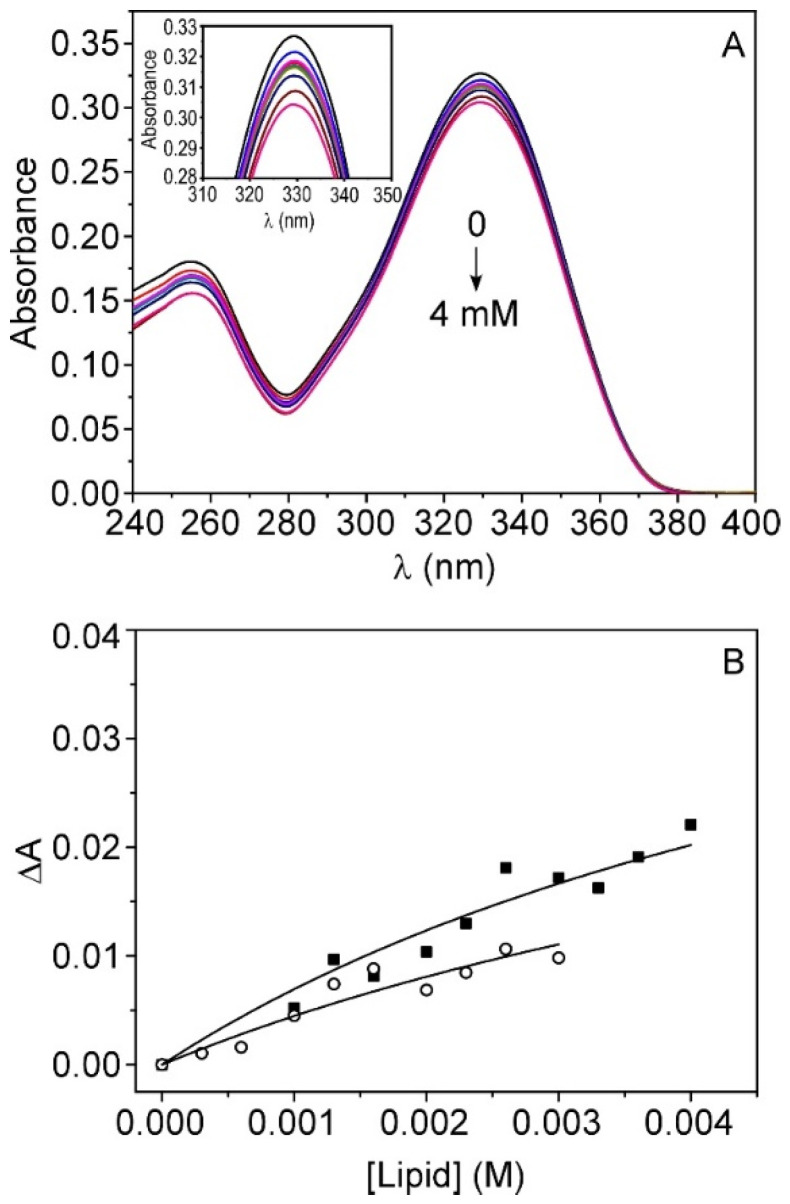
(**A**) Absorption spectrum of TMZ (150 µM) in water in presence of egg 1,2-diacyl-sn-glycero-3-phosphocholine (EPC) Large unilamellar vesicles (LUVs), at increasing lipid concentrations, up to 4 mM. Inset: Amplified image. (**B**) Changes observed in the absorbance values of TMZ at 329 nm (∆A), at increasing EPC (black square) and EPC:Chol (3:1) (circle) lipid concentration. Solid lines represent fitting curves to Equation (8).

The interaction of TMZ with blood cells was assessed using simplified model membranes that simulate the lipid composition of the human erythrocyte plasma membrane. For this purpose, LUVs composed of phosphatidylcholine without and with cholesterol (3:1), which are among the most abundant lipid species in the outer membrane leaflet of human erythrocytes, were obtained [58]. Because membrane lipids are not fluorescent, the interaction of TMZ with the different LUVs was first explored by recording the absorption spectra of the drug (150 µM) at increasing concentrations of lipid. For these experiments, 0.2 cm path length cuvettes were used, in order to minimize scattering background signals, and TMZ was dissolved in water at 25 °C to avoid its possible degradation during the measurements. Figure 2A shows the absorption spectra of TMZ in EPC LUVs recorded at different lipid concentrations, after the subtraction of lipid contribution. The addition of the LUVs decreased the absorbance of TMZ very slightly and gradually, while maintaining the shape of the spectrum, which discards drug degradation and suggests some interaction between the drug and the lipid membrane. The small decrease in absorbance could be explained by the existence in solution of two stable rotamers of TMZ [59]. Recently, Khallilian et al. reported that the rotation of carboxamide moiety in TMZ leads to two rotamers with slight differences in their extinction coefficients and dipole moments; Rotamer 1 being more stable than Rotamer 2 [60]. The interaction of TMZ with the lipid membrane could stabilize Rotamer 2, which exhibits a lower extinction coefficient than Rotamer 1. Similar results were found for samples containing LUVs from EPC and cholesterol, as it is shown in Supporting Information (Figure S1).

In an attempt to estimate the affinity of the drug for the lipid membrane, the partition coefficient, *K*_p_, of TMZ between lipid vesicles and aqueous medium was calculated from the changes observed in the absorbance values at 329 nm (∆A), plotting ∆A versus [lipid], and fitting these data to Equation (8) [61]:

∆A = (∆A_max_[lipid])/(1/((*K*_p_γ) + [lipid])) (8)

In this equation, γ = 0.9 M^−1^ is the molar volume of the lipid [62,63] and ∆A_max_ is the difference between the absorbance of TMZ in the absence of LUVs (A_TMZ_) and the absorbance obtained when all the TMZ is bound to the lipid vesicles (A_TMZ -LUVs_). In order to facilitate the fit, A_TMZ -LUVs_ was fixed, assuming that the extinction coefficient of TMZ in membrane is close to that corresponding to Rotamer 2 [60]. The solid lines in Figure 2B show these fits, which yield values of *K*_p_ = 159 ± 10 and *K*_p_ = 133 ± 16 for EPC and EPC:Chol, respectively. These parameters are one order of magnitude lower than those obtained recently by Ramalho et al. for TMZ and DMPC [18]. For this reason, a second estimation of *K*_p_ was performed, allowing A_TMZ -LUVs_ to be free, but the error corresponding to its determination was so large that it was only possible to conclude that the value of *K*_p_ was below 1000 in both EPC and EPC:Chol membranes. We also tried to estimate *K*_p_ representing the second derivative of the absorbance, instead of the absorbance values, as suggested by Kitamura et al. [64], but it was not possible to improve the uncertainty of its determination. In any case, these results suggest that TMZ has a very low affinity to red cell plasma membranes, at least to the lipid component of these membranes.

**Figure 3:**
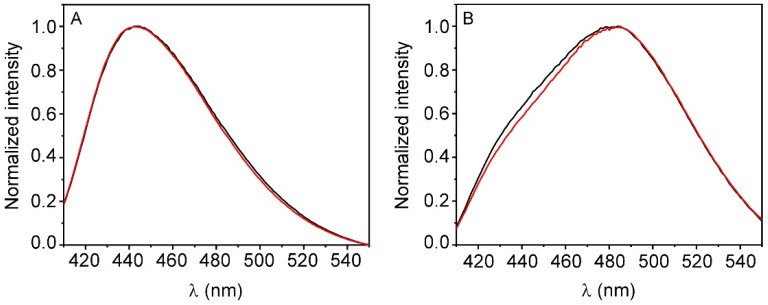
Fluorescence emission spectra of Laurdan (λexc = 390 nm) in 1,2-dimyristoyl-sn-glycero-3-phosphocholine (DMPC) LUVs (0.5 mM) in water before (black) and after addition (red) of TMZ (150 µM), at 15 °C (**A**) and 37 °C (**B**).

Indirect measurements were also performed in order to obtain more insight on the TMZ-lipid membrane interactions, using two fluorescent reporter molecules: Laurdan and DPH. For these experiments, DMPC LUVs (0.5 mM) were prepared instead to EPC LUVs to additionally explore the effect of TMZ on the gel-to-fluid lipid phase transition, which takes place at 23 °C. Laurdan is a fluorescent probe with a high dipole moment in the excited state as compared to the ground state. Therefore, its fluorescence spectrum strongly depends on the rate of dipolar relaxation, as well as on the hydrophilic/hydrophobic character of its surrounding environment [65,66,67]. Once incorporated in the bilayer, the probe localizes close to the surface, at the level of the glycerol backbone of the phospholipids [68]. The disordered conformation of lipids, characteristic of the fluid phase, favors the motions and permeability of water molecules into the bilayer, so the Laurdan emission spectrum shifts to the red, from 440 nm (gel phase) to 490 nm (fluid phase). This property makes the probe sensitive to changes in the membrane lipid packing [65,66,67]. Figure 3A,B show the emission spectrum of Laurdan in DMPC (0.5 mM) in water at 15 °C (gel phase) and 37 °C (fluid phase), before and after the addition of TMZ (150 µM). The results show that, for the probe in the gel phase, the emission spectrum was unmodified after the addition of TMZ, while in the fluid phase, the emission band was slightly narrower, and the intensity observed around 440 nm decreased with respect to that obtained in the absence of TMZ. The addition of higher concentrations of the drug did not induce any further change in the spectrum. This suggests that, in spite of its low affinity, the binding of TMZ to the fluid phase membrane probably weakens the interactions between the lipid polar heads, enhancing the permeability of water molecules into the bilayer. Therefore, the drug should be preferentially located in the membrane/water interfacial region.

Unlike Laurdan, the fluorescent probe DPH incorporates into the membrane at the nonpolar environment, mostly parallel to the lipid chains. Its steady-state anisotropy, <r>, simultaneously contains structural and dynamics information on the more hydrophobic region of the bilayer. Therefore, to explore whether the interaction of TMZ with DMPC LUVs also affects the physical properties of this region, the anisotropy of DPH was determined at 15 °C (gel phase) and 37 °C (fluid phase), before and after the addition of the drug. In the absence of TMZ, a high value of <r> = 0.339 was found at 15 °C, as expected for motion-restricted environments. This value decreased to 0.092 during the fluid phase, evidencing a strong increase in the conformational freedom of the lipid hydrocarbon chains, occurring above the transition temperature. Addition of TMZ (100 and 150 µM) to the LUVs did not alter these values (Table 1), supporting the idea that TMZ should partition into the membrane polar–apolar interfacial region in preference to the hydrophobic core of the bilayer.

This conclusion was also supported from the study of the thermotropic behavior of DMPC in the absence and presence of TMZ (100 and 150 µM). The plot of the anisotropy of DPH versus temperature is a common tool used to characterize this behavior. Figure S2 shows the changes in <r> recorded for the different samples. The shape of the plot was perfectly sigmoidal in the three systems, with a sharp drop of anisotropy around 23 °C, being coincident with the phase transition temperature of DMPC. This result is indicative of the preservation of the integrity of the hydrophobic core of the lipid bilayer, after the binding of the drug, evidencing that TMZ is not embedded into the bilayer. A similar experiment was carried out by considerably increasing the concentration of TMZ in the sample, up to 5 mM. Results were identical, as above, indicating that even at this concentration TMZ does not penetrating deeply into the bilayer.

Finally, we explore whether the binding of TMZ to the lipid membrane could delay the decomposition process of TMZ to MTIC and AIC. These experiments were made in phosphate buffer pH 7.4 at 25 °C, to compare the results with those obtained with free TMZ. Following the same steps, the absorption spectra of a sample containing TMZ (150 µM) and EPC (3 mM) were recorded as a function of time (Figure S3). Results were practically identical to those obtained for free TMZ, indicating that the binding of TMZ to the lipid vesicles does not delay the hydrolysis process, and therefore it does not stabilize the drug. Because TMZ degradation commences by adding a water molecule to carbonyl moiety (in red in Scheme 1), the findings suggest that this group remains completely exposed to the water environment and, therefore, the drug is not embedded into the bilayer, as supported from previous Laurdan and DPH experiments. Overall, it can be inferred that the interaction of the TMZ with lipid membranes is practically negligible, thus, very little impact is expected on the bioavailability of the drug in plasma, due to membrane-lipid binding. In addition, these results suggest that complexation of TMZ with erythrocytes is not an adequate alternative to improve its administration. The low affinity of TMZ to the lipid bilayer is likely associated with the high number of hydrogen bond acceptors (two carbonyl groups and six nitrogen atoms) of the molecule. Recently, Kasende et al. have reported the high tendency of TMZ to form H-bonds with water molecules, leading to stable complexes of different geometries [69]. In addition, the same group have shown that TMZ can form homodimers with other TMZ molecules in water, stabilized by H-bonds or involved in π-stacking interactions [70].

### 3.3. Interaction of TMZ with HSA

**Figure 4:**
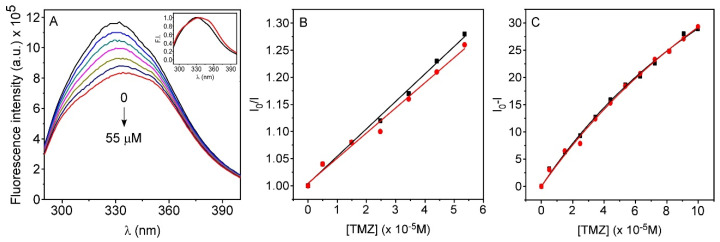
(**A**) Fluorescent emission spectra of human serum albumin (has) (6.2 µM) (λ_exc_ = 280 nm) in water at 25 °C, and at increasing concentrations of TMZ. Inset: Normalized emission spectra of HSA in the absence (black) and the presence (red) of TMZ (55 µM). (**B**) Stern–Volmer plots for quenching of HSA by TMZ in water (black) and sodium phosphate buffer pH 7.4 (red). (**C**) Plot of ∆I = (I_0_-I) versus [TMZ] in water (black) and sodium phosphate buffer pH 7.4 (red).

The interaction of TMZ with HSA was monitored from changes observed in the intrinsic fluorescence of the protein. Experiments were carried out, in the first instance, in water, to minimize the degradation of TMZ during the measurements. HSA has a sole tryptophan residue, Trp-214, located in a hydrophobic pocket in subdomain IIA, which is mainly responsible for its intrinsic fluorescence. The protein also has 18 tyrosine residues (Tyr) distributed along the whole polypeptide chain. Upon excitation at 280 nm, both Trp and Tyr are excited, but most of the fluorescence comes from Trp-214, due to the energy transfer from tyrosine to tryptophan [46,71]. Figure 4A shows the emission spectrum of HSA recorded at 25 °C. The spectrum displays a broad fluorescence band with a maximum at 334 nm, which indicates that Trp-214 is relatively buried inside the protein, in accordance with the crystallographic data. Figure 4A also shows the spectra of HSA recorded after increasing concentrations of TMZ, from 0 to 55 µM, once corrected according to Equation (1) for primary and secondary inner-filter effects. The addition of TMZ induced a gradual decrease in the protein fluorescence intensity, accompanied with a slight alteration in the shape of the spectrum (Inset in Figure 4A), which evidence the existence of interactions between drug and protein. From the inset, it is possible to appreciate that, at high TMZ concentrations, the emission due to Tyr residues, which takes place at 306 nm, is slightly more pronounced, suggesting that the Trp fluorescence is especially quenched by TMZ, unmasking the fluorescence of some of the tyrosines. In addition, a shoulder appears to longer wavelengths (≈350 nm), suggesting either that there is a decrease in the hydrophobicity surrounding Trp-214 after TMZ binding, or that the drug induces a conformational change in the protein.

Stern–Volmer plots, which show the relation between relative fluorescence intensities I_0_/I of the protein (calculated as the area under the spectra) and the molar TMZ concentration, are shown in Figure 4B. For all the investigated concentrations, the Stern−Volmer plot exhibited a good linear relationship. The Stern–Volmer constant, *K*_SV_ (Equation (3)), obtained from the slope of the plot, is shown in Table 2. From these values, a bimolecular quenching rate constant, *k*_q_ = 8.2 × 10^11^ M^−1^ s^−1^ was estimated, assuming τ_0_= 6.4 ns [46]. This value is higher than the maximum diffusion collision rate constant of various quenchers with the macromolecule (2.0 × 10^10^ M^−1^ s^−1^), suggesting that the quenching mechanism is mainly static, and that a ground-state complex is formed between the drug and the protein [72].

As can be observed in Figure S4, there is a good overlap region between the fluorescence spectrum of HSA and the absorption spectrum of TMZ. This suggests that the decrease in fluorescence, induced by the formation of the drug-protein complex, could be in part attributed to a fluorescence resonance energy transfer (FRET) from the singlet excited state of tryptophan to TMZ. This process is not uncommon, and has been reported in other occasions for the ensemble of HSA and its ligands [73,74]. In order to evaluate this possibility, the critical energy-transfer distance R_0_ (Förster radius) was calculated using Equation (6). From the spectral overlap between HSA emission and TMZ absorption bands, the overlap integral J for the TMZ-HSA complex was found to be 6.68 × 10^13^ M^−1^ cm^−1^ nm^4^. By taking a value of quantum yield of 0.1 [71], a value of R*_0_* ≈ 21 Å was obtained, which is lower than that of ≈70 Å, corresponding to the hydrodynamic diameter of HSA [75]. Because the energy transfer process is only significant at distances r < 1.5R*_0_* [71], we can conclude that, once bound to HSA, the average distance between TMZ and Trp-214 is ≤ 31 Å. While it might be possible to obtain the value of r with more precision, by determining the energy transfer efficiency, this information would not be reliable, because FRET is probably not the only mechanism involved in the fluorescence quenching of HSA. 

To estimate the binding constant (*K*_a_) between TMZ and HSA, we assumed the simplest of situations, which is the formation of a 1:1 complex. Given the structure of TMZ, it is very probable that more than one protein site is involved in the formation of the complex, but we assume affinities rather similar for any of them. The most generally valid equation to analyze changes in fluorescence upon formation of this type of complexes can be significantly simplified to Equation (4), if the added drug concentration is larger than the protein concentration [47]. For this reason, higher concentrations of TMZ were added to has, and the resultant fluorescence intensities were fitted to that equation (Figure 4C). From this fit, a *K*a = 5103 ± 58 M^−1^ (dissociation constant, *K*d ≈ 0.2 mM) was obtained, which suggests a moderate affinity of the TMZ for the plasma protein. The residual fluorescence (Ic) obtained from the fit was close to zero. This result indicates that the fluorescence of the fully saturated protein should be totally quenched, suggesting that one of the binding protein sites of TMZ is located in domain II of HSA, very close to Trp-214.

It is possible to get more insight into the nature of this interaction by determining the thermodynamic parameters (Δ*G*, Δ*H* and Δ*S*) of the binding reaction. The binding forces between small drugs and proteins include electrostatics, Van der Waals, H-bonding and hydrophobic interactions. In general, according to the rules summarized by Ross and Subramanian [76], an endothermic process, together with an important increase in entropy, suggest that the hydrophobic interaction plays the major role in this association, while small or negative ΔS indicate Van der Waals and H-bonds. ΔG, ΔH and ΔS can be estimated using the Vant´Hoff equation, Equation (5), if the binding constant is obtained at more than one temperature. Table 2 shows *K*_SV_ and *K*a values obtained for the TMZ-HSA interaction at 15 °C, and the thermodynamics parameters determined from this equation. Higher temperatures were not explored, due to the instability of TMZ shown in Figure 1B. From Table 2, it can be seen that for TMZ-HSA complex, ΔG and ΔH, are negative, indicating that TMZ can spontaneously bind with HSA through an exothermic process. In addition, for the binding process an entropy value of 57 J mol^−1^ K^−1^ was determined. Altogether, these results suggest that far beyond hydrophobic interactions, Van der Waals forces and H-bonds play a leading role in the binding process. In fact, some of the proton bond acceptors of TMZ could establish hydrogen bonds with donor atoms of the protein amino acids. In addition, π-stacking interactions could probably occur between the aromatic ring systems of TMZ and Trp-214, which would explain the total quenching of the fully saturated protein previously discussed. 

The above experiments were made in water to avoid the hydrolytic instability of TMZ at neutral pH. However, to assess if the binding process is modified under physiological conditions, we performed the experiments in phosphate buffer, pH 7.4, in samples containing has, and increasing concentrations of TMZ. The stability of the drug during the experiment was controlled from its absorption spectrum, taking into account the results shown in Figure 1. As in water, the fluorescence of the protein decreased with the addition of the drug. The Stern–Volmer plot was linear (Figure 4B) and a value of *K*_SV_ = 4837 ± 243 M^−1^ was obtained from the slope, which was very similar to that registered in water at the same temperature (25 ºC). The binding constant and free energy change were determined as previously described, from the fit of data to Equation (4) (Figure 4C), and using Equation (5). Values of *K*a = 4016 ± 758 M^−1^ (*K*d ≈ 0.25 mM) and ΔG = −2.06 × 10^4^ J mol^−1^ were obtained, which were also very close to those found in water, evidencing that the binding process is practically not affected by the presence of the buffer. 

### 3.4. Interaction of TMZ with AGP

**Figure 5:**
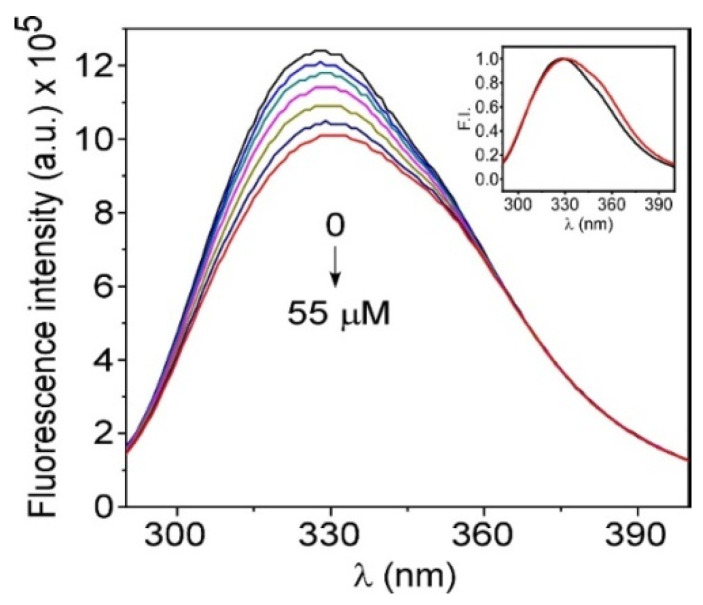
Fluorescence emission spectra of AGP (6.2 µM) (λ_exc_ = 280 nm) in water at 25 °C, with increasing concentrations of TMZ. Inset: Normalized emission spectra of AGP in the absence (black) and the presence (red) of TMZ (55 µM).

As for HSA, we studied the affinity of TMZ by AGP, another important protein on human plasma. Changes in its intrinsic fluorescence upon the addition of TMZ were used to estimate the binding affinity. The fluorescence of this protein is contributed by three Trp residues in positions 25, 122 and 160 [41]. Trp-25 is buried in the hydrophobic pocket, while Trp-122 lies between the pocket and the protein surface, and Trp-160 is totally exposed to the surface. Figure 5 shows the fluorescence spectra of AGP obtained at different TMZ concentrations, after inner filter correction. Results show that the fluorescence intensity of the protein is quenched by the drug, as was observed for HSA (Figure 4A), but the quenching process is slightly less efficient. The shape of the spectrum was also altered (Inset in Figure 5), but to a lesser extent than that of HSA. From the fit of these data to a Stern–Volmer plot, values of *K*_SV_= 3093 ± 127 M^−1^ and *K*q = 4.83 x 10^−11^ M^−1^ s^−1^ were obtained (Table 2 and Figure S5A), evidencing that a static mode of fluorescence quenching is operative, and that, as for HSA, a ground state complex is formed between the anticancer drug and the protein.

The binding constant *K*a and free energy change ΔG of the process were determined as previously described, from the fit of data to Equation (4) and Equation (5) (Figure S5B). Values of *K*a = 8184 ± 983 M^−1^ and ΔG = −2.23 × 10^4^ J mol^−1^ were determined, which indicate that TMZ also spontaneously binds with AGP to form the complex (Table 2). It is interesting to note that, in spite of the *K*_SV_ value being lower than that determined for HSA, the *K*a is slightly higher in AGP. This suggests that the three tryptophans of this protein are not quenched with the same efficiency, which is also supported by the fact that a residual fluorescence (Ic) was obtained from the fit of data to Equation (4), whose value was around 30% of the uncomplexed protein fluorescence. As for HSA, the decrease in fluorescence could be in part attributed to a FRET process between the tryptophans of AGP and the drug. From the spectral overlap between the protein emission and TMZ absorption bands, the overlap integral J for the TMZ-AGP complex was found to be 7.17 × 10^13^ M^−1^ cm^−1^ nm^4^. By taking a value of quantum yield of 0.07 (determined in our laboratory at λexc = 280 nm, using N-acetyltryptophanamide, NATA, as reference), the calculated R_0_ value was ≈20.7 Å, which is lower than the size of the protein and supports, as for HSA, the possible existence of FRET process after the complex formation. 

As mentioned in the Introduction section, the concentration of HSA in blood is clearly higher (more than one order of magnitude) than that of AGP. Therefore, although the affinity constant is slightly higher for AGP than for HSA, we can conclude that, in human plasma, most of the bound drug will be associated with HSA. In fact, under ideal conditions, in the absence of competitive ligands, up to ≈68% of the total TMZ present in blood could be bound to HSA, while AGP binds only ≈2%, according to the *K*a values determined and the reported HSA, AGP and TMZ blood concentrations [22].

Taking into account the moderate binding constant of the TMZ-HSA complex, together with the previously commented long half-life of HSA in serum (four times longer than the AGP) and the overexpression of albumin-binding receptors on tumor cells, it would not be unreasonable to think that a part of the administered TMZ is transported through the bloodstream by the HSA to the GBM cells. Recent studies have reported physical changes in the blood-brain barrier, in the case of brain metastases [16]. These morphological alterations are linked to molecular alterations, which are responsible for an increase in the permeability of this barrier, allowing the albumin to pass through this compartment, carrying the host drug into the brain.

Additional studies were therefore undertaken, in order to gain more insight into the formation of this complex. 

### 3.5. Stability of TMZ-HSA Complex

**Figure 6:**
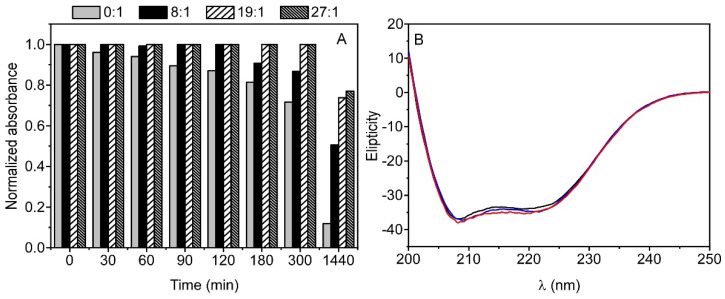
(**A**) Bar diagrams showing the normalized maximum absorption of TMZ (26 µM) in phosphate buffer pH 7.4 at 25°C as a function of time, in absence (gray) and in presence of increasing HSA:TMZ molar ratios; 8:1 (black), 19:1 (striped) and 27:1 (densely striped). (**B**) Effect of TMZ on the CD spectrum of HSA (3.0 μM). TMZ concentrations were: 0 (black), 20 (blue) and 200 μM (red).

We first explored whether the interaction of TMZ with HSA had any effect on its kinetics of decomposition into MTIC and AIC. To check this possibility, a series of samples containing TMZ (26 µM) and increasing concentrations of HSA were prepared in phosphate buffer pH 7.4 and their absorption spectra were recorded at 25 °C, as a function of time. Figure 6A shows the evolution of the normalized absorption maximum of the drug measured at predetermined intervals of time for each of these samples. The results show that the hydrolysis phenomenon is delayed as the HSA concentration increases, and therefore the amount of TMZ bound to it is greater (from 0 to ≈73%, using *K*a value in buffer). This enhancement in stability confirm the formation of the complex and suggests that the carbonyl moiety of the molecule (in red in Scheme 1) is less accessible to water molecules once bound to the protein, probably because it is located in a more hydrophobic region and/or involved in hydrogen bonding with amino acids. This result supports the previous suggestion of TMZ interacting with Trp-214 in domain II of HSA. The complexation of TMZ to other macromolecules and nanoparticles has also been reported to enhance drug stability, with decomposition kinetics slower than that of free TMZ [1,77,78]. This result could explain the different decomposition half-lives reported in the literature, for TMZ at 37°C in buffer (1 h) and blood plasma (1.8 h) [3,6]. The enhancement observed in plasma may be due to the interaction of TMZ with the plasma proteins, as demonstrated here, and with other components which protect the drug from the external environment. 

### 3.6. CD Measurements on the TMZ-HSA Complex

In order to study whether the formation of the TMZ-HSA complex destabilizes the protein, as was previously suggested, CD spectra were recorded in the absence and presence of increasing concentrations of the drug. Figure 6B shows the far-UV CD spectrum of HSA (3 µM), alone, and after the addition of TMZ (20 µM and 200 µM). In the absence of the drug, HSA exhibited two negative bands at 208 nm and 222 nm, which are characteristic peaks of the alpha-helical structure of proteins. The addition of TMZ practically did not modify the signal of HSA, even at 200 µm, supporting that the protein conformation is still essentially the same after complex formation. 

### 3.7. Molecular Docking and Dynamics Simulations of TMZ-HSA Complex

Ligand-protein interactions can modulate the activity of numerous targets involved in the biological processes of medical interest. While molecular docking generates a static picture of the recognition of a ligand by a binding site in the protein, the computational technique of molecular dynamics (MD) simulates the dynamic behavior of the molecular system (ligand, receptor, water and ions) as a function of time. Occasionally, the predictions of molecular docking on a potential modulator of a protein are not confirmed by MD data. Therefore, both techniques should be used when we intend to predict the experimental behavior of a ligand. HSA structures co-crystallized with TMZ are not available, so we have carried out molecular docking simulations to understand, in molecular terms, the TMZ-HSA interaction observed in our experimental data.

**Figure 7:**
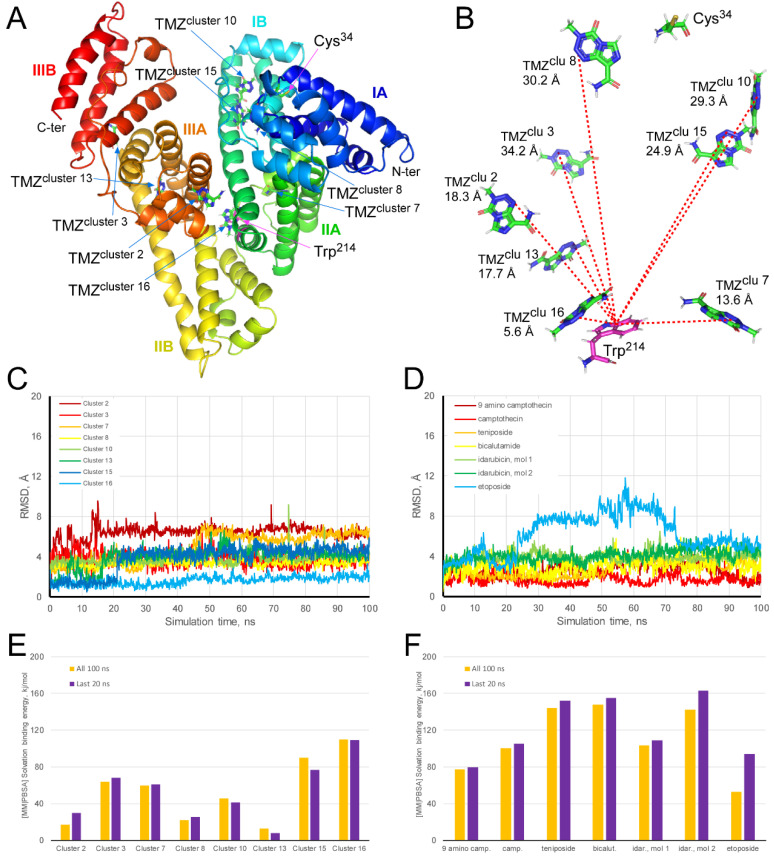
Molecular dynamics simulation of HSA-TMZ complex throughout 100 ns. (**A**) Secondary structure of HSA in rainbow color, from N-terminal (blue) to C-terminal (red). The three subdomains (I, II and III), as well as the amino acids Trp-214 and Cys-34, have been indicated. The docked TMZ molecule with the lowest Gibbs free energy of each cluster has been shown, indicating its location with blue arrows. (**B**) Distance (Å) between Trp-214 and the TMZ molecules of each cluster. (**C**,**D**) The trajectory (RMSD, Å) of TMZ molecules docked to HSA and different antineoplastic drugs bound to HSA, respectively. Molecular mechanics/Poisson–Boltzmann surface area solvation binding energy analysis of the HSA forming a complex with TMZ (**E**) and some antineoplastic (**F**), using YASARA dynamics v19.9.17 software. The best-docked complex, as the initial conformation for MD simulation, followed by 1000 snapshots (100 ns) obtained from the MD trajectory, were employed to calculate the values of solvation binding energy of TMZ or antineoplastic drugs. Additionally, the average value calculated for the last 200 snapshots (20 ns) is also displayed. YASARA-calculated binding energy provides positive values when the predicted binding is strong and stable whereas, negative values indicate no binding. Figure 7A,B were prepared using PyMol 2.3 software.

The results of molecular docking simulations predict the existence of up to 16 highly populated clusters of TMZ docked to HSA. The dissociation constants (*K*d) calculated from these data range from submilimolar to millimolar values (0.016 to 0.233 mM, Supporting Information Table S1). Figure 7A,B show the location and distance (Å) of the TMZ clusters in relation to the Trp-214 and Cys-34 residues. Only those clusters in which, after 100 ns of molecular dynamics simulation, the TMZ molecule remains bound to has, are shown (Figure 7B,C and Figure S6). Only eight of the initial sixteen clusters remain bound, and in several clusters, the Gibbs free energy variation (ΔG, J mol^−1^, Supporting Information Table S1) is even less detached from the protein throughout the MD time. The results of both molecular docking and MD predict that cluster 16 of TMZ (Figure 7 and Figure S6) would establish π-stacking interactions with Trp-214, supporting the hypothesis previously proposed. In fact, the values of ΔG= −2.07 × 10^4^ J mol^−1^ and *K*a = 4292 M^−1^ (*K*d = 0.233 mM) calculated for this cluster agree very well with those obtained from fluorescence quenching experiments (Table 2). Several TMZ molecules detached from HSA during MD simulation adopt a stacked conformation in aqueous solution (data not shown), in agreement with the results reported by Kasende et al. [70] 

To further verify the stability of the HSA-TMZ complexes, we carried out an evaluation of the molecular mechanics/Poisson–Boltzmann surface area (MM/PBSA) parameter [79]. MM/PBSA estimates the free energy of the binding of small ligands to biological macromolecules and uses MD simulations of the receptor-ligand complex. It is known that MM|PBSA show good correlations with the values obtained experimentally, despite excluding the conformational entropy or the number and free energy of water molecules in the binding site [79,80]. Figure 7E shows the MM|PBSA average binding energy (calculated as the average of the last 20 ns, or during the 100 ns of total duration of the MD) of TMZ clusters that remain attached to HSA after 100 ns of MD simulation. Three aspects should be highlighted in these results, (i) for all TMZ clusters, the average value calculated for the 100 ns is very similar to that calculated for the last 20 ns. (ii) TMZ clusters 15 and 16 show the highest values (≈83 and ≈104 kJ mol^−1^, respectively, even though they showed the less negative ΔG values (Supporting Information Table S1). (iii) MM|PBSA values for clusters 15 and 16 are similar to those calculated for several antineoplastic drugs such as 9-aminocamptothecin (≈29 kJ mol^−1^), idem camptothecin (≈121 kJ mol^−1^,), bicalutamide (≈146 kJ mol^−1^) idarubicin (≈104 for molecule 1 and ≈146 for molecule 2, kJ mol^−1^), teniposide (≈146 kJ mol^−1^), and etoposide (≈67 kJ mol^−1^) (Figure 7D,F). The values of MM|PBSA solvation binding energy over 83–125 kJ mol^−1^ can be considered to be very moderate if we compare them with values over 1040 kJ mol^−1^ for the binding of 17β-estradiol to the estrogen receptor ligand binding domain (manuscript in preparation). Therefore, these studies reveal different binding site for this drug, and suggest that TMZ is mainly bound to the subdomain IIA (cluster 16) and subdomain IB (cluster 15) of HSA, with similar *K*d and MM|PBSA values. These binding sites, especially subdomain IB, have been reported for other antineoplasic drugs, such as the previously mentioned camptothecin, idarubicin, teniposide or bicalutamide [33]. These predictions support the experimental results well: TMZ in subdomain IIA is mainly responsible for the protein fluorescence quenching, while its location in subdomain IB, being about 25Å away from the Trp-214 (Figure 7B), has much less effect on the fluorescence intensity, as the probability of FRET is much lower. The fact that the hydrolysis phenomenon is delayed in the TMZ-HSA complex can also be understood from the detailed map of molecular interactions depicted in Figure S6. In cluster 16, TMZ is inserted into a hydrophobic cavity, establishing π-stacking interactions with Trp-214, so the carbonyl moiety of the drug is practically inaccessible to water molecules. In cluster 15, the OH group of Tyr138 is connected through a hydrogen bond to the carbonyl moiety of TMZ, protecting the molecule from the hydrolysis process. 

In conclusion, we can consider that the data provided from the computational approach to the study of the TMZ-HSA interaction support the idea that albumin is a transporter with moderate affinity for TMZ, which travels through the blood plasma protected in the hydrophobic pockets of the protein.

## 4. Conclusions

The interaction of the alkylating antineoplasic agent temozolomide (TMZ) with blood components has been explored, to better understand how the drug travels through the bloodstream. TMZ displays an almost negligible interaction with model membranes (EPC and EPC:Chol) that mimic the lipid content of the outer membrane of erythrocytes. The calculated *K*p values are below 1000 in the two lipid systems studied, and the drug is not able to penetrate into the membrane, remaining exposed to the water environment and interacting only with the membrane surface. Such interactions should not, therefore, have a noticeable effect on the drug bioavailability.

Binding studies with plasma proteins, in particular with human serum albumin (HSA) and alpha-1-acid glycoprotein (AGP), reveal a moderate affinity of TMZ for these proteins, with *K*a values in the range of 5–8 × 10^3^ M^−1^ in water. Due to the differences between the two proteins, namely, the concentration of HSA is more than one order of magnitude higher than that of AGP under physiological conditions, and its serum half-life is four times longer—these results suggest that, in human plasma, most of the bound TMZ will be attached to HSA rather than to AGP. 

The nature of the binding forces in TMZ-HSA conjugates is likely based on Van der Waals interactions and H-bond formation that add up to hydrophobic forces and probably π-stacking, as is suggested by the determined thermodynamic parameters and the molecular docking predictions. These interactions do not affect the protein conformation, but increase the hydrolytic stability of the drug under physiological conditions, slowing down its degradation kinetics.

From the results of the present study, together with the reported overexpression of albumin-binding receptors on tumor cells, we suggest that a remarkable part of the orally administered TMZ could reach the target site (i.e., GBM cells) protected in the hydrophobic pockets of HSA. 

This study not only contributes to the further understanding of the bioavailability of TMZ, but can also be used to design more efficient formulations, which increase the stability and therapeutic index of the drug, a task that has been pursued by a large number of laboratories for years [1,12,81,82,83,84,85,86]. In this regard, HSA-based nanocarriers seem to be suitable candidates for designing biomimetic delivery systems that selectively transport TMZ to GBM cells. 

## Figures and Tables

**Table 1:** 1-6-diphenylhexatriene (DPH) steady state anisotropy values, <r>, in DMPC liposomes at different TMZ concentrations.

TMZ (μM)	<r> (15 °C) ± SD	<r> (37 °C) ± SD
0	0.339 ± 0.001	0.092 ± 0.002
100	0.339 ± 0.003	0.091 ± 0.002
150	0.340 ± 0.001	0.093 ± 0.001

**Table 2:** Stern–Volmer constants (*K*_SV_), binding parameters (*K*a) and thermodynamic parameters (ΔG, ΔH, ΔS) of HSA-TMZ and AGP-TMZ systems in water at different temperatures.

		Stern–Volmer Constants	Binding Parameter	Thermodynamic Parameters		
Protein	T(K)	*K*_SV_ (M^−1^) ^a^	*K*a (M^−1^) ^a^	ΔG (J mol^−1^) ^a^	ΔH (J mol^−1^) ^a^	ΔS (J mol^−1^K^−1^) ^a^
HSA	288	4462 ± 334	5442 ± 140	−2.06 × 10^4^ ± 62	−4571 ± 2644	57 ± 9
	298	5232 ± 245	5103 ± 58	−2.11 × 10^4^ ± 28		
AGP	298	3093 ± 127	8184 ± 983	−2.23 × 10^4^ ± 298		

^a^ ± SD.

## Supplementary Materials

The following are available online at https://www.mdpi.com/2218-273X/10/7/1015/s1, Figure S1: Effect of EPC:Chol on the TMZ absorption spectrum; Figure S2: Temperature curves for DPH fluorescence anisotropy within DMPC LUVs in presence and absence of TMZ; Figure S3: Influence of EPC LUVs on TMZ degradation as a function of time; Figure S4: Overlap between HSA fluorescence and TMZ absorption; Figure S5: Fittings of AGP fluorescence quenching by TMZ to Stern–Volmer and ΔI curves; Figure S6: Map of TMZ molecular interactions in each cluster at different times of molecular dynamics simulation; Table S1: Details of the interaction of TMZ docked to HSA (ΔG, *K*d, n).
