# Supplementary material for: The Interaction of Temozolomide with Blood Components Suggests the Potential Use of Human Serum Albumin as a Biomimetic Carrier for the Drug
Source: Biomolecules. 2020 Jul 9;10(7):1015. doi: 10.3390/biom10071015 (PMC7408562; doi:10.3390/biom10071015)
Supplement: Supplementary file 1 [file biomolecules-10-01015-s001.pdf]

Supporting information

The Interaction of Temozolomide with Blood Components Suggests the Potential Use of Human Serum Albumin as a Biomimetic Carrier for the Drug

Marta Rubio-Camacho, José A. Encinar, María José Martínez-Tomé, Rocío Esquembre and C. Reyes Mateo**

Instituto de Investigación, Desarrollo e Innovación en Biotecnología Sanitaria de Elche (IDiBE), Universidad Miguel Hernández (UMH), E-03202 Elche, Spain.

***Corresponding authors:** resquembre@umh.es, rmateo@umh.es

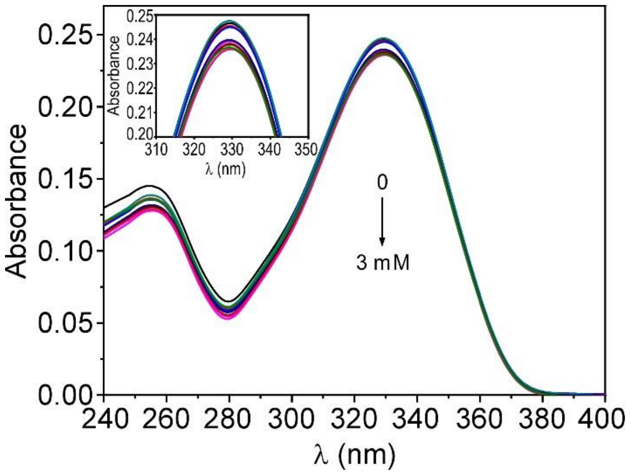

Figure S1. Absorption spectrum of TMZ (150 μM) in water in presence of EPC:Chol (3:1) LUVs, at increasing lipid concentrations, up to 3 mM. Inset: Amplified image.

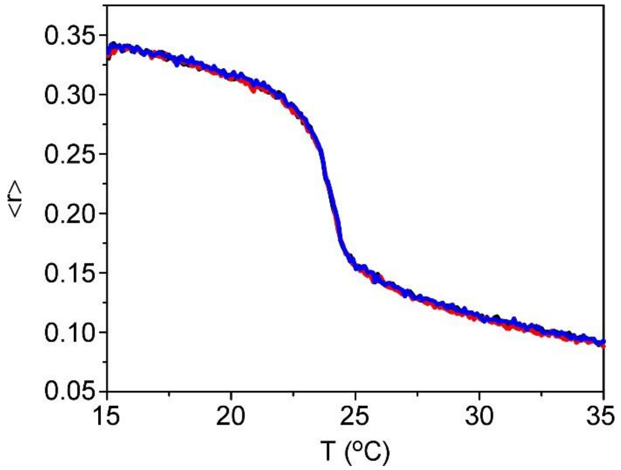

Figure S2. Effect of temperature on the fluorescence anisotropy <r> of DPH in DMPC LUVs (0.5 mM), in absence (black) and presence of TMZ 100 μM (blue).and 150 μM (red).

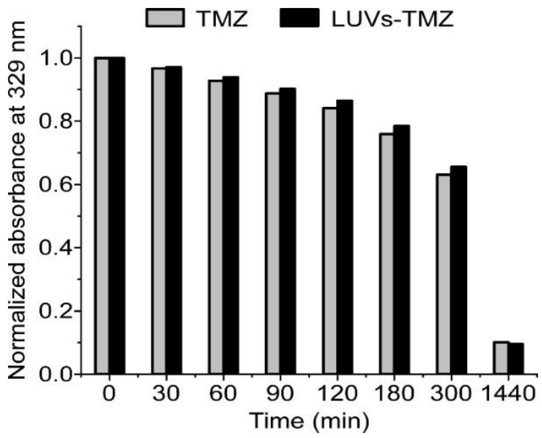

Figure S3. Bar diagrams showing the normalized maximum absorption of TMZ (150 μM) in phosphate buffer at 25ºC in absence (gray) and presence (black) of EPC LUVs (3 mM), as a function of time.

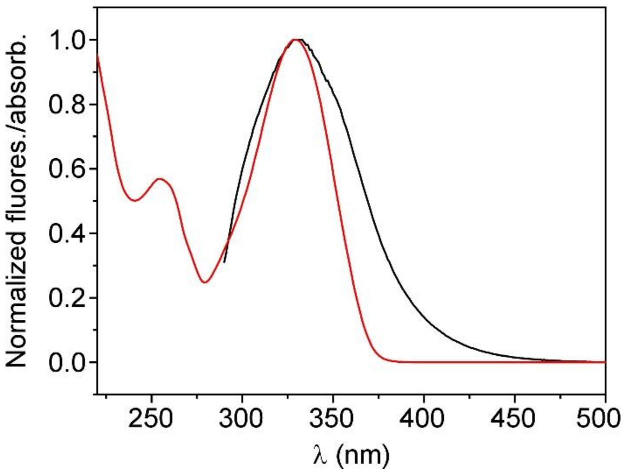

Figure S4. Overlap between the fluorescence emission spectrum of HSA (black) (λ_{exc}= 280 nm) and the absorption spectrum of TMZ (red).

.

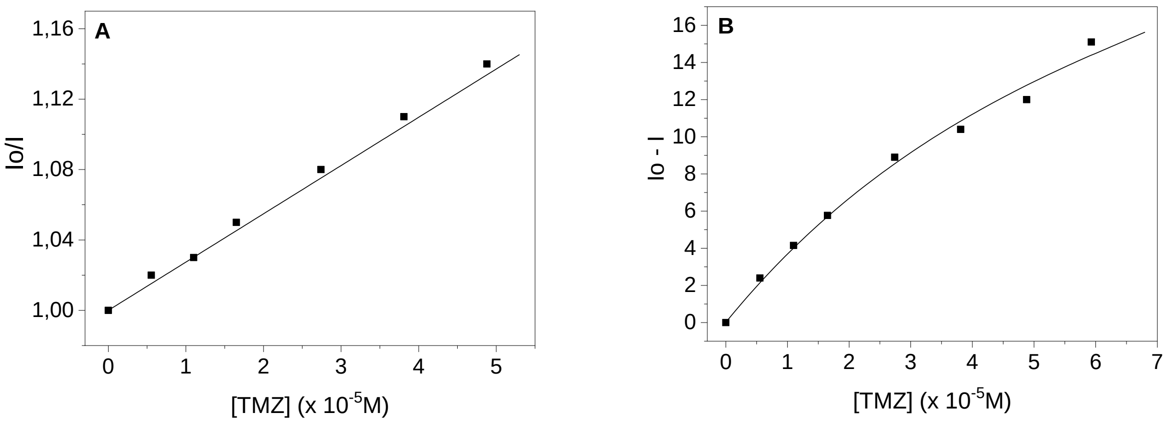

Figure S5. (A) Stern-Volmer plot for quenching of AGP fluorescence by TMZ in water. (B) Variations of AGP fluorescence, $\Delta I = (I_0 - I)$ at increasing concentration of TMZ.

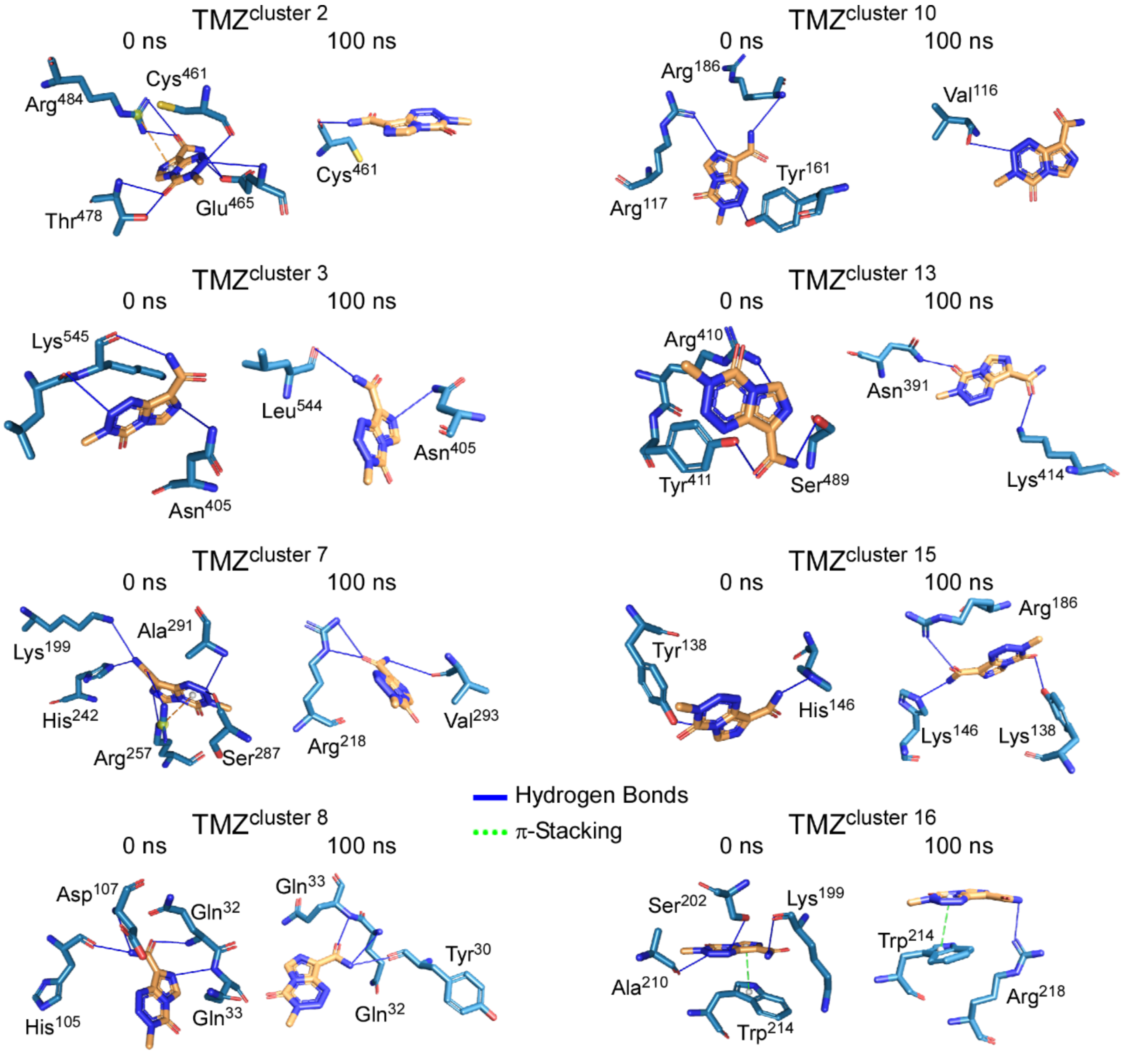

Figure S6. A detailed map of the molecular interactions of TMZ in each cluster before (0 ns) and after 100 ns of molecular dynamics simulation. Each inset shows the detailed interactions of each TMZ cluster docked to HSA using the PLIP algorithm [50], indicating the participating amino acids involved in the interaction and the type of interaction (hydrogen bonds, salt bridges, π-stacking, etc). Figures were prepared using PyMol 2.3 software.

Table S1. Details of the interaction of TMZ docked to HSA. (UniProt code: P02768, PDB code: 1AO6). For the best-docked TMZ molecule of each cluster, the Gibbs free energy (ΔG, J mol^{-1}), the dissociation constant, and the number of molecules members (as %) are shown. For docked-TMZ molecules that have remained bound to HSA after 100 ns of molecular dynamics simulation the parameters shown in this table have been highlighted in bold.

**Cluster number**	**ΔG (J mol^{-1}) · 10^4**	**K_d [mM]**	**Members, %**
1	-2.742	0.016	2.90
**2**	**-2.571**	**0.031**	**2.70**
**3**	**-2.541**	**0.035**	**3.20**
4	-2.462	0.042	3.90
5	-2.462	0.048	1.50
6	-2.374	0.069	2.30
**7**	**-2.303**	**0.091**	**1.60**
**8**	**-2.282**	**0.100**	**1.70**
9	-2.270	0.104	0.70
**10**	**-2.249**	**0.114**	**1.80**
11	-2.245	0.116	1.30
12	-2.199	0.140	1.40
**13**	**-2.132**	**0.182**	**1.30**
14	-2.115	0.196	1.00
**15**	**-2.098**	**0.210**	**0.80**
**16**	**-2.073**	**0.233**	**0.90**
